# An open-label, 1-year extension study of the long-term safety and efficacy of once-daily OROS^® ^hydromorphone in patients with chronic cancer pain

**DOI:** 10.1186/1472-684X-8-14

**Published:** 2009-09-15

**Authors:** Magdi Hanna, Alberto Tuca, John Thipphawong

**Affiliations:** 1 Director of Pain Research Unit, King's College Hospital, King's College London, UK; 2 Analgesics & Pain Research, 62 Park Rd, Beckenham, Kent, UK; 3 Medical Coordinator, Hospital Support Team of Palliative Care, Hospital Duran i Reynals, Instituto Catalán de Oncología, Av. Gran Vía de L'Hospitalet 199-203, L'Hospitalet, Barcelona, Spain; 4 Johnson and Johnson Pharmaceutical Research Division Corporation, 6500 Paseo Padre Boulevard, Fremont, California 94555, USA

## Abstract

**Background:**

Opioid analgesics have proven efficacy in the short-term management of chronic cancer pain, but data on their long-term use is more limited. OROS^® ^hydromorphone is a controlled-release formulation of oral hydromorphone that may be particularly well suited to long-term management of chronic cancer pain because it provides stable plasma concentrations and consistent analgesia with convenient once-daily dosing. The objective of this study (DO-118X) was to characterise the pain control achieved with long-term repeated dosing of OROS^® ^hydromorphone in patients with chronic cancer pain.

**Methods:**

In this multicentre, phase III, open-label, single treatment, 1-year extension study, OROS^® ^hydromorphone was administered to 68 patients with moderate-to-severe chronic cancer pain, who had successfully completed a short-term equivalence study, and whose pain was controlled with a stable dose of medication (≥ 8 mg OROS^® ^hydromorphone or equivalent controlled-release morphine). Patients were started on the dose of OROS^® ^hydromorphone equivalent to the opioid dose on which they achieved dose-stable pain control in the equivalence study; dose adjustments were made as necessary and breakthrough pain medication was permitted. Efficacy was assessed with the Brief Pain Inventory (BPI) and patient and investigator global evaluations of treatment effectiveness. No formal statistical analysis was done.

**Results:**

The mean (standard deviation) duration of exposure to study medication was 139 (129.9) days and the mean (standard deviation) average daily consumption of OROS^® ^hydromorphone was 43.7 (28.14) mg/day. All scores were maintained at a mild to moderate severity throughout the study; however, BPI scores for pain at its worst, pain at its least, pain on average, pain right now, and pain relief were slightly worsened at end point compared with baseline. Mean BPI pain interference with daily activities and patient and investigator global evaluation scores also remained generally stable. Treatment effectiveness was rated as fair to good throughout the study. The most frequently reported adverse events were nausea (n = 24, 35.3%), constipation (n = 22, 32.4%), and vomiting (n = 15, 22.1%).

**Conclusion:**

The results of this extension study suggest that long-term repeated dosing with once-daily OROS^® ^hydromorphone can be beneficial in the continuing management of persistent, moderate-to-severe cancer pain.

## Background

Chronic severe pain is a common complication of cancer [1]. Opioid analgesics are highly effective at treating cancer pain and are typically used after maximum doses of non-opioid analgesics have failed [2-5]. The European Association for Palliative Care [6] and the American Pain Society [7] support the use of long-term analgesics for maintaining pain relief once individual dose requirements have been established.

Hydromorphone hydrochloride is a hydrogenated semi-synthetic potent μ-opioid agonist that has been used for many years to treat moderate-to-severe cancer pain. Numerous studies have demonstrated an efficacy and safety profile similar to that of morphine and other opioids [8-10]. For oral administration, it is available as short-acting immediate-release (IR) and long-acting controlled-release (CR). OROS^® ^hydromorphone (Jurnista™; Janssen Pharmaceutica, Beerse, Belgium) is a novel, once-daily, CR formulation of oral hydromorphone which uses a patented Push-Pull™ osmotically-controlled pump system (ALZA Corporation, Mountain View, CA, USA) to release hydromorphone in a continuous monophasic manner for up to 24 hours. It is an important treatment option for patients with chronic pain as it provides consistent pain relief, convenient once-daily dosing, and can reduce opioid-related adverse effects and breakthrough pain associated with peak and trough fluctuations in plasma concentrations typically seen with IR formulations [11,12]. OROS^® ^hydromorphone is currently available in 4, 8, 16, 32, and 64 mg tablets.

The pharmacokinetic (PK) properties of OROS^® ^hydromorphone demonstrate that hydromorphone is released in a consistent manner from the dosage form. Plasma hydromorphone concentrations peak significantly later (12-18.0 hours versus 0.8 hours) but are maintained significantly longer at greater than 50% of peak concentration (22.7 hours versus 1.1 hours) with OROS^® ^hydromorphone than with IR hydromorphone [13]. The plasma concentrations achieved after OROS^® ^hydromorphone administration reach approximately 80% of the peak concentration within 6-8 hours and remain elevated until approximately 18-24 hours post-dose [12]. The mean absolute bioavailability of hydromorphone after a single dose of 8, 16, or 32 mg of OROS^® ^hydromorphone ranged from 22% to 26%. Clinical PK analysis has shown a consistent release of hydromorphone over 24 hours, with steady-state plasma concentrations achieved by 48 hours (2 doses) and sustained throughout the 24-hour dosing interval [14,15]. Further research has confirmed that the PK of OROS^® ^hydromorphone are linear and dose-proportional across the available doses [16]. The apparent terminal half-life of OROS^® ^hydromorphone is 10-11 hours [16]. A close relationship between plasma concentration and analgesic activity has been described for OROS^® ^hydromorphone [13].

An osmotically-controlled system means that release of the drug from the system is not significantly affected by environmental factors such as pH or gastric motility [17]. There is a minimal effect of food on the rate and extent of absorption of hydromorphone from OROS^® ^hydromorphone [18], and the PK are not significantly affected by alcohol, with no evidence of 'dose dumping' of hydromorphone [19]. In addition, conversion from previous standard opioid therapy to OROS^® ^hydromorphone can be achieved without loss of pain control or increase in adverse events (AEs) in patients with chronic malignant [20,21] and non-malignant pain [22].

The safety and tolerability of hydromorphone is well established, with a side effect profile similar to that of other opioid analgesics (mild to moderate constipation, dizziness, nausea, and vomiting). Analyses of the oral IR formulation in special populations concluded that gender does not affect the PK of hydromorphone [23]; however, mean peak concentration (C_max_) was decreased by 14% and overall exposure (AUC) was increased by 11% in elderly (aged 65-74 years) compared with younger (aged 18-38 years) patients receiving single doses of hydromorphone [24]. For this reason, it is advised that the treatment of the elderly with hydromorphone should be cautious and the initial dose should be reduced [15]. Hydromorphone has been found to be safe and effective in patients with impaired renal or hepatic function, although it is advised to be used with caution and close monitoring owing to the increased exposure to (mean C_max _and AUC were 2- to 4-fold higher) and slower elimination of hydromorphone and its metabolites in these patients [25-29].

Glucuronidation is the main metabolic pathway of hydromorphone and the principal metabolite is hydromorphone-3-glucuronide. It is unlikely that hydromorphone would be involved in drug interactions involving cytochrome P450 (CYP) because studies have shown hydromorphone is metabolised via non-CYP dependent pathways and only minimally metabolised by P450 enzymes [30,31]. Hydromorphone also lacks the analgesically active metabolites of many opioids that may lead to respiratory depression if accumulated and demonstrates a very low plasma protein binding (< 30%) [32,33]. For these reasons OROS^® ^hydromorphone may be especially suitable and predictable for elderly patients, patients with renal or hepatic insufficiency, and patients with multiple morbidities and medications.

Two recent studies have compared OROS^® ^hydromorphone to other commonly used opioid analgesics: CR morphine [34] and extended-release (ER) oxycodone [35]. In patients with cancer pain, clinical equivalence in terms of Brief Pain Inventory (BPI) scores for 'worst pain in the past 24 hours' was not demonstrated for OROS^® ^hydromorphone and CR morphine. However, the negative direction of the mean difference between the treatments was in favour of OROS^® ^hydromorphone and comparable results were found for secondary efficacy measures such as assessments of pain interference with daily activities [34]. With OROS^® ^hydromorphone, pain intensity scores were similar in the morning and evening (measured by BPI pain now AM and PM), and pain levels in the evening were significantly lower with OROS^® ^hydromorphone compared with CR morphine. This confirms that OROS^® ^hydromorphone provides consistent pain relief over 24 hours and that there is little end-of-dose failure pain. The half value duration (the time period in which the plasma level of the active ingredient is over the half-maximum concentration) can be used to measure the prolongation of the duration of action of CR preparations and therefore test for end-of-dose failure pain; the half value duration of OROS^® ^hydromorphone is between 27 and 29 hours [36]. In the second comparative study, once-daily OROS^® ^hydromorphone and twice-daily ER oxycodone provided comparable levels of pain relief and reductions in pain severity, as well as improvements in investigator and patient global evaluation scores and subjective measures of daily function and sleep, in patients with chronic, moderate to severe osteoarthritis pain [35]. In both studies, AEs were comparable between treatments and typical of opioid analgesic therapy.

Although opioids have proven efficacy in the management of chronic moderate-to-severe pain, data on their long-term use is limited, as most research has used relatively short-term studies [37-39]. This issue has become progressively more important in recent years as the life expectancy of cancer patients increases owing to improved oncological therapies. As a result, long-term opioid use in cancer patients has become widespread, and therefore data on the safety and efficacy of long-term exposure is necessary [37-42]. This study was an extension study for patients successfully completing a previous equivalence study, which was a randomised, double blind study to test the clinical equivalence of IR and CR formulations of hydromorphone and morphine in 200 adult patients with chronic moderate-to-severe cancer pain [34]. The primary objective of this extension study was to characterise the pain control achieved with long-term repeated dosing, for up to 1 year, of OROS^® ^hydromorphone in patients with chronic cancer pain.

## Methods

The study (DO-118X) was approved by the independent ethics committee appropriate to each participating centre before any patients were enrolled at that centre, and was conducted in accordance with the recommendations of the Declaration of Helsinki and the European Community Commission Directive 91/507/EEC by adopting the Good Clinical Practice (GCP) principles as defined in the International Conference on Harmonisation (ICH) guidelines for GCP (CPMP/ICH/135/95). All patients gave written informed consent before entering the study.

### Patients

The study enrolled adult (≥ 18 years of age) patients with chronic cancer pain, who had completed the randomised, double blind equivalence study, and whose pain was controlled with a stable dose of study medication, ≥ 8 mg/day of either OROS^® ^hydromorphone or an equivalent CR morphine sulphate dose, during the final 2 days of the CR phase of the equivalence study. The criteria used for patient selection are listed in Table 1. It was planned to include up to 140 patients.

**Table 1:** Criteria for patient selection

**Inclusion criteria**	**Exclusion criteria**
Patients with chronic cancer pain who had successfully completed the previous equivalence study. Notably, patients were required to have been in dose-stable pain control in the last 2 days of the CR phase of the study	Pure or predominantly neuropathic pain or pain of unknown origin (where a mechanism or physical cause could not be identified)
Patients requiring at least 8 mg of hydromorphone every 24 hours for the management of chronic cancer pain	A recent (within the previous 6 months) or current history of drug and/or alcohol abuse
Written informed consent	Women of childbearing potential who were pregnant or lactating, seeking pregnancy, or failing to take adequate contraceptive precautions (i.e. abstinence, an oral contraceptive, a hormonal implant, an intrauterine device, or condoms/diaphragm and spermicide)
	Intolerance of or hypersensitivity to hydromorphone or other opioids
	Dysphagia
	Vomiting judged by the investigator sufficient to interfere with oral analgesia
	Any gastrointestinal disorder (except gastrointestinal cancers), including pre-existing severe gastrointestinal narrowing (pathologic or iatrogenic) that may have affected the absorption or transit of orally administered drugs, particularly the insoluble OROS^® ^outer coating
	Acute abdominal conditions that may have been obscured by opioids
	Any significant central nervous system disorder including, but not limited to head injury, increased intracranial pressure, stroke within the previous 6 months, major clinical depression, and disorders of cognition which, in the opinion of the investigator, would interfere with the completion of patient assessments and study compliance (patients with stable cerebral metastases could be included)
	Risk of serious decreases in blood pressure upon administration of an opioid analgesic (e.g. depleted blood volume, comprised vasomotor tone, circulatory shock)
	Severe respiratory compromise or severely depressed ventilatory function, impaired renal or hepatic function, Addison's disease, hypothyroidism, prostatic hypertrophy, or urethral stricture which in the opinion of the investigator precluded the use of strong opioids
	Receiving or received mono-amine oxidase inhibitors within the previous 2 weeks
	Those previously entered into the study
	Participation in another study with an investigational drug in the previous 4 weeks, or an analgesia study within the previous 8 weeks (with the exception of the equivalence study)

### Study design

This was a phase III, multicentre, open-label, single treatment arm, 1-year extension study. It was conducted at 17 centres in Europe and Canada.

The screening process for patients entering the study was their participation in and completion of the previous equivalence study. Patients then completed a baseline visit (visit 1), which was also the final visit in the equivalence study, during which, the inclusion and exclusion criteria were reviewed, a physical examination was done, the BPI was administered, and the study drug was dispensed.

All patients received the same treatment, OROS^® ^hydromorphone. Patients were started on a dose of OROS^® ^hydromorphone equivalent to the opioid dose on which they had achieved dose-stable pain control in the CR phase of the equivalence study (using a 5:1 conversion ratio of morphine sulphate to hydromorphone hydrochloride [43-46]). Dose adjustments, to be made after 2 days of therapy at a dose level, were then made as needed, based on the patient's degree of opioid tolerance, general condition and medical status, concurrent medication, type and severity of pain, and the amount and frequency of rescue medication needed for breakthrough pain. Dose increases were to be generally in 8 mg increments for patients receiving total daily doses of up to 32 mg and 16 mg increments in patients receiving doses of greater than 32 mg/day. IR hydromorphone 2 and 4 mg tablets were dispensed for breakthrough pain. The maximum daily dose of rescue medication was not to exceed 10-15% of the daily OROS^® ^hydromorphone dosage.

The treatment phase of the study lasted for up to 1 year, during which time patients returned to the clinic at monthly intervals for assessment. During these monthly evaluations, any unused study medication was collected and new medication was dispensed, the BPI and global evaluations of overall medication effectiveness were administered, and AEs and concomitant medications were documented. Patients were able to receive a bowel regimen for the management of chronic opioid-related constipation if necessary.

At 12 months or premature discontinuation (when a patient discontinued from the study early), the study completion visit was carried out. At this visit, the BPI and global evaluations were administered, AEs and concomitant medications were documented, and a physical examination was done.

### Statistical methods

All data from patients who had received at least 1 dose of study medication were included in all efficacy and safety analyses.

The primary efficacy measure was 5 questions of the BPI assessing pain qualities in the past 7 days [47], which was completed by the investigator in consultation with the patient at baseline, each monthly visit, and study completion or early discontinuation. The following BPI end points were investigated:

• Change from baseline in pain at its worst in the past 7 days (BPI question 3)

• Change from baseline in pain at its least in the past 7 days (BPI question 4)

• Change from baseline in pain on average (BPI question 5)

• Change from baseline in current pain (BPI question 6)

• Change from baseline in pain relief in the past 7 days (BPI question 8)

BPI questions 3, 4, 5, and 6 were measured on a scale of 0 (no pain) to 10 (pain as bad as you can imagine); question 8 was measured on a scale of 0% (no relief) to 100% (complete relief).

Secondary efficacy measures were assessed monthly and at study completion or early discontinuation. The first secondary efficacy measure was an evaluation of quality of life (QoL) from question 9 of the BPI, analysed as change from baseline in how pain has interfered with the patient's life in the past 7 days. This question included 7 subsections: general activity, mood, walking ability, normal work, relationships, sleep, and enjoyment of life, which were measured on a scale of 0 (does not interfere) to 10 (completely interferes). Another secondary efficacy measure was a global assessment of overall treatment effectiveness completed by both the patient and the investigator separately. This was measured on a 5-point scale (1 = poor, 2 = fair, 3 = good, 4 = very good, 5 = excellent).

No formal statistical testing was done on the data; only summary statistics were produced. For baseline and each of the 12 monthly visits, absolute observed values were used; whereas end point was calculated using the last observation carried forward (LOCF) method.

Safety measures included monitoring of AEs, early discontinuations, concomitant medications, and physical examination findings. GCP standards were followed to record all AEs occurring during the study regardless of whether they were considered to be related to the study drug. Formal definitions of AEs and questionnaires were not used. AEs were coded using the Medical Dictionary for Regulatory Activities (MedDRA) classification. Safety data were summarised descriptively.

## Results

### Study population

68 patients were enrolled into the study (Belgium, n = 14; Canada, n = 7; France, n = 2; Germany, n = 2; Netherlands, n = 22; Spain, n = 7; UK, n = 14). 35 patients had been taking OROS^® ^hydromorphone and 33 had been taking CR morphine sulphate in the previous equivalence study. 10 patients (14.7%) completed the 1-year study, 4 patients (11.4%) who had previously been taking OROS^® ^hydromorphone and 6 patients (18.2%) who had been taking CR morphine. The reasons for not completing the study are shown in Table 2; the most common reasons for not completing were death (22.1% of patients) and progression of disease (20.6%). Only a small proportion discontinued owing to lack of efficacy (11.8%). The rate and reasons for the dropouts did not appear to be related to prior therapy. The baseline demographics and clinical characteristics of the study population were similar between patients who had taken the two previous treatments (Table 3).

**Table 2:** Patient disposition (overall and by previous treatment)

	**Treatment in previous study**		
**Number (%)**	**OROS^**® **^hydromorphone**	**CR morphine**	**Overall**
Entered the study	35 (100)	33 (100)	68 (100)
Completed the study	4 (11.4)	6 (18.2)	10 (14.7)
Did not complete study	31 (88.6)	27 (81.8)	58 (85.3)
**Reasons for not completing the study**			
Death	10 (28.6)	5 (15.2)	15 (22.1)
Progression of study disease	7 (20.0)	7 (21.2)	14 (20.6)
Adverse event	5 (14.3)	4 (12.1)	9 (13.2)
Lack of efficacy	4 (11.4)	4 (12.1)	8 (11.8)
Protocol violation	3 (8.6)	4 (12.1)	7 (10.3)
Withdrawal of consent	1 (2.9)	2 (6.1)	3 (4.4)
Administrative reason	1 (2.9)	1 (3.0)	2 (2.9)

CR, controlled-release

**Table 3:** Baseline demographic and clinical characteristics (overall and by previous treatment)

	**Treatment in previous study**		
**Variable**	**OROS^**® **^hydromorphone** **(n = 35)**	**CR morphine** **(n = 33)**	**Overall** **(n = 68)**
Mean (SD) age, years	57.4 (15.24)	58.3 (10.05)	57.8 (12.89)
Sex, n (%)			
Male	9 (25.7)	16 (48.5)	25 (36.8)
Female	26 (74.3)	17 (51.5)	43 (63.2)
Race, n (%)			
White	35 (100.0)	32 (97.0)	67 (98.5)
Asian	0	1 (3.0)	1 (1.5)
Mean (SD) height, cm	165.9 (8.32)	168.2 (10.76)	167.0 (9.58)
Mean (SD) weight, kg	62.4 (16.09)	68.0 (9.90)	65.1 (13.60)
Mean (SD) BMI, kg/m^2^	22.8 (5.90)	24.2 (4.18)	23.5 (5.14)
Cancer type, n (%)			
Breast	11 (31.4)	12 (36.4)	23 (33.8)
Gastrointestinal	9 (25.7)	5 (15.2)	14 (20.6)
Genitourinary	5 (14.3)	3 (9.1)	8 (11.8)
Lung	5 (14.3)	7 (21.2)	12 (17.6)
Oral cavity	1 (2.9)	1 (3.0)	2 (2.9)
Other	4 (11.4)	5 (15.2)	9 (13.2)
Location of metastases, n (%)^1^	57 (100)	58 (100)	115 (100)
Bone	16 (28.1)	20 (34.5)	36 (31.3)
Bone marrow	1 (1.8)	1 (1.7)	2 (1.7)
Brain	1 (1.8)	2 (3.4)	3 (2.6)
Kidney	1 (1.8)	0	1 (0.9)
Liver	9 (15.8)	9 (15.5)	18 (15.7)
Lung	6 (10.5)	6 (10.3)	12 (10.4)
Lymph node	8 (14.0)	6 (10.3)	14 (12.2)
None	4 (7.0)	7 (12.1)	11 (9.6)
Other	11 (19.3)	7 (12.1)	18 (15.7)
Predominant pain type, n (%)			
Bone or soft tissue	22 (62.9)	26 (78.8)	48 (70.6)
Mixed	6 (17.1)	3 (9.1)	9 (13.2)
Visceral	7 (20.0)	4 (12.1)	11 (16.2)

^1^shows total number of metastases; patients may be counted in more than one metastasis location

BMI, body mass index; CR, controlled-release; SD, standard deviation

### Extent of exposure to study medication

During the study, for all patients, the mean (standard deviation [SD]) duration of exposure to study medication was 139 (129.9) days (range, 2.0 to 438 days). The mean (SD) average daily consumption of OROS^® ^hydromorphone was 43.7 (28.14) mg/day (range, 9.6 to 139.2 mg/day). Each of these variables was slightly higher in patients who received morphine in the previous study.

At the beginning of the study, 8 patients received doses of ≥ 64 mg (64 mg, n = 1; 72 mg, n = 4; 96 mg, n = 3); at end point, this increased to 20 patients (64 mg, n = 2; 72 mg, n = 4; 80 mg, n = 3; 96 mg, n = 6; and n = 1 each for 112, 128, 168, 176, and 192 mg).

### Efficacy results

#### Primary efficacy end point

**Figure 1:**
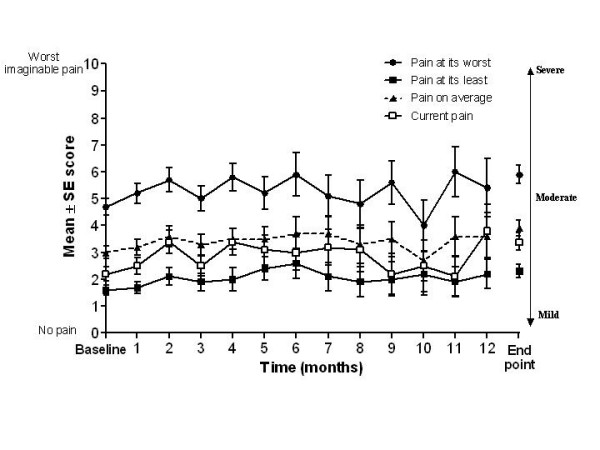
**BPI questions 3, 4, 5, and 6: summary from baseline to subsequent months and end point (all patients)**. Baseline and months 1 to 12 = absolute values; end point = last observation carried forward. Participating patient numbers - pain at its worst: n = 65 at baseline, n = 10 at month 12, and n = 67 at end point; pain at its least: n = 66 at baseline, n = 10 at month 12, and n = 67 at end point; pain on average: n = 66 at baseline, n = 10 at month 12, and n = 67 at end point; current pain: n = 66 at baseline, n = 10 at month 12, and n = 67 at end point. BPI, brief pain inventory; SE, standard error.

**Figure 2:**
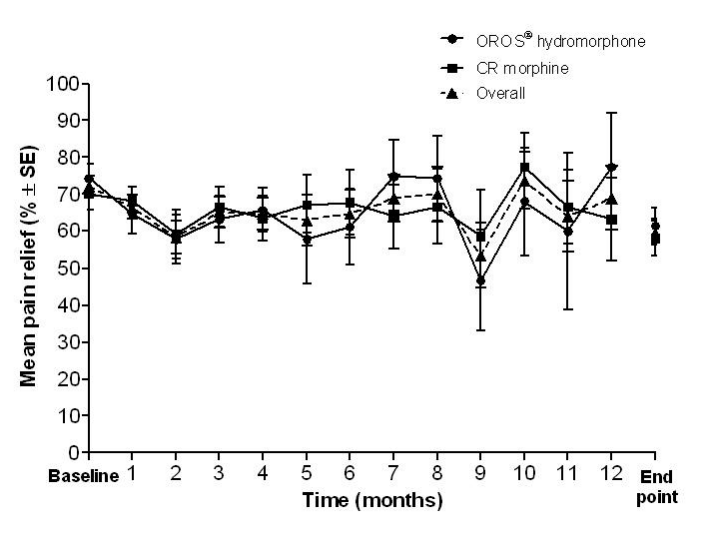
**BPI pain relief: summary from baseline to subsequent months and end point (overall and by previous treatment)**. Baseline and months 1 to 12 = absolute values; end point = last observation carried forward. Participating patient numbers - OROS^® ^hydromorphone: n = 32 at baseline, n = 4 at month 12, and n = 33 at end point; CR morphine: n = 32 at baseline, n = 6 at month 12, and n = 33 at end point; overall: n = 64 at baseline, n = 10 at month 12, and n = 66 at end point. BPI, brief pain inventory; CR, controlled-release; SE, standard error.

Pain control was maintained during the year with repeated once-daily dosing of OROS^® ^hydromorphone. Table 4 shows the mean scores at baseline and end point for BPI items pain at its worst, pain at its least, pain on average, pain right now, and pain relief. When looking at the change in scores during the course of the study, pain at its least, pain on average, and current pain scores were maintained at a mild severity and worst pain scores fluctuated between mild and moderate severity throughout the 1-year study (Figure 1). Although scores were maintained at similar levels throughout the study, the mean scores were slightly increased, i.e. worsened, at end point compared with baseline. Pain relief also remained fairly stable throughout the study (Figure 2), with mean (SD) scores of 72.2% (22.8%) and 59.8% (27.6%) at baseline and end point, respectively.

**Table 4:** BPI scores at baseline and end point (overall and by previous treatment)

	**Treatment in previous study**		
**BPI variable** **Mean (SD)**	**OROS^**® **^hydromorphone** **(n = 35)**	**CR morphine** **(n = 33)**	**Overall** **(n = 68)**

Pain at its worst^1^			
Baseline	4.3 (2.30)	5.1 (2.78)	4.7 (2.57)
End point	5.9 (2.58)	5.8 (2.98)	5.9 (2.76)
Pain at its least^1^			
Baseline	1.3 (1.23)	1.8 (1.91)	1.6 (1.62)
End point	2.2 (1.81)	2.4 (2.43)	2.3 (2.12)
Pain on average^1^			
Baseline	2.8 (1.89)	3.2 (2.10)	3.0 (2.00)
End point	3.9 (2.30)	3.8 (2.50)	3.9 (2.38)
Current pain^1^			
Baseline	1.8 (1.84)	2.5 (2.21)	2.2 (2.05)
End point	3.3 (2.86)	3.5 (2.49)	3.4 (2.67)
Pain relief^2^			
Baseline	74.4 (22.13)	70.0 (23.56)	72.2 (22.78)
End point	61.5 (27.40)	58.2 (28.11)	59.8 (27.60)

Baseline = absolute values; end point = last observation carried forward

^1^0 = no pain, 10 = pain as bad as you can imagine; ^2^0% to 100%

BPI, brief pain inventory; CR, controlled-release; SD, standard deviation

#### Secondary efficacy end points

**Figure 3:**
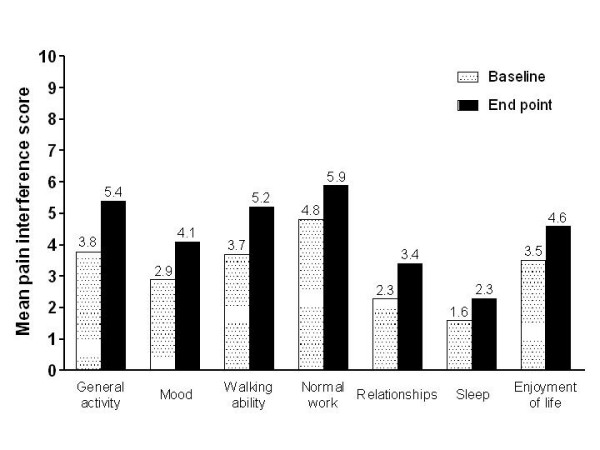
**BPI pain interference scores at baseline and end point (all patients)**. BPI scored from 0 = does not interfere to 10 = completely interferes. BPI, brief pain inventory.

Mean BPI pain interference scores remained stable during the study, increasing only slightly from baseline to end point for each of the QoL items (general activity, mood, walking ability, normal work, relationships, sleep, and enjoyment of life). BPI pain interference scores at baseline and end point for all patients are shown in Figure 3. The pain interference results by treatment in the previous equivalence study generally reflected the overall results and there were no major differences between patients who had previously received OROS^® ^hydromorphone and CR morphine.

**Figure 4:**
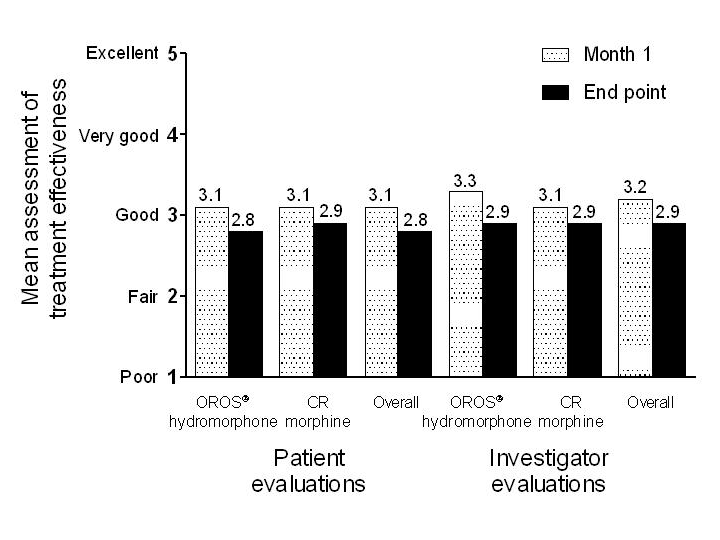
**Patient and investigator global evaluations at month 1 and end point (overall and by previous treatment)**. Scale: 0 = poor, 2 = fair, 3 = good, 4 = very good, 5 = excellent. CR, controlled-release.

Mean patient and investigator global evaluation scores of overall treatment effectiveness also remained generally stable from baseline to end point (Figure 4). Treatment effectiveness was rated as fair to good throughout the study.

### Safety results

Overall, 63 patients (92.6%) reported AEs during the study, 34 patients (97.1%) who had received OROS^® ^hydromorphone in the previous equivalence study and 29 patients (87.9%) who had received CR morphine. The most commonly reported AEs, with at least a 10% incidence, were nausea, constipation, vomiting, anaemia, peripheral oedema, dyspnoea, asthenia, disease progression, somnolence, and urinary tract infection. AEs reported in at least 5% of patients are shown in Table 5.

**Table 5:** Adverse events reported by at least 5% of patients (overall and by previous treatment)

	**Treatment in previous study**		
	**OROS^**® **^hydromorphone** **(n = 35)**	**CR morphine** **(n = 33)**	**Overall** **(n = 68)**
**Adverse event**	**Number (%) of patients with adverse event**		
Nausea	13 (37.1)	11 (33.3)	24 (35.3)
Constipation	13 (37.1)	9 (27.3)	22 (32.4)
Vomiting	8 (22.9)	7 (21.2)	15 (22.1)
Anaemia	7 (20.0)	5 (15.2)	12 (17.6)
Peripheral oedema	5 (14.3)	6 (18.2)	11 (16.2)
Dyspnoea	4 (11.4)	5 (15.2)	9 (13.2)
Asthenia	4 (11.4)	5 (15.2)	9 (13.2)
Somnolence	6 (17.1)	2 (6.1)	8 (11.8)
Disease progression	6 (17.1)	2 (6.1)	8 (11.8)
Urinary tract infection	4 (11.4)	3 (9.1)	7 (10.3)
Headache	4 (11.4)	2 (6.1)	6 (8.8)
Diarrhoea	5 (14.3)	1 (3.0)	6 (8.8)
Back pain	4 (11.4)	1 (3.0)	5 (7.4)
Anorexia	3 (8.6)	2 (6.1)	5 (7.4)
Oedema	3 (8.6)	2 (6.1)	5 (7.4)
Dehydration	0	5 (15.2)	5 (7.4)
Confusional state	4 (11.4)	1 (3.0)	5 (7.4)
Pyrexia	4 (11.4)	1 (3.0)	5 (7.4)
Pain	1 (2.9)	4 (12.1)	5 (7.4)
Insomnia	1 (2.9)	3 (9.1)	4 (5.9)
Dry mouth	2 (5.7)	2 (6.1)	4 (5.9)
Anxiety	3 (8.6)	1 (3.0)	4 (5.9)

CR, controlled-release

Most AEs were considered mild or moderate in severity, approximately 25% of all reported AEs were considered severe. Severe AEs were reported by 43 patients (63.2%), and severe AEs considered related to study treatment were reported by 11 patients (16.2%). 36 patients (52.9%) reported AEs that were considered related to study treatment (20 patients [57.1%] who had received OROS^® ^hydromorphone in the previous equivalence study and 16 patients [48.5%] who had received CR morphine). Of the most common AEs (= 10% incidence in a treatment group), some cases of nausea (11/24), constipation (18/22), vomiting (7/15), and somnolence (6/8) were considered related to study treatment. In addition, all cases of dry mouth were considered related to study treatment as well as several cases of confusional state, anxiety, and insomnia. None of the reports of diarrhoea or headache was considered related to study treatment.

19 patients died either during or after the study. The relationship to treatment of the AEs leading to death was considered unlikely in 2 cases and unrelated in the other 17 cases. In addition to the deaths, other serious adverse events (SAEs) were reported by 32 patients, the majority of which were considered unrelated or unlikely to be related to study medication. 8 patients (11.8%) had SAEs that were considered to be possibly, probably, or definitely related to study treatment; these were: nausea and vomiting in 2 patients; dehydration, malaise, nausea (2 episodes), pain, and vomiting (2 episodes) in 1 patient; faecaloma in 1 patient; dizziness and nausea in 1 patient; restlessness in 1 patient; suicide attempt in 1 patient; and confusional state, hallucination, and pain in 1 patient. The faecaloma and suicide attempt events were considered to have a definite relationship to study treatment.

9 patients (13.2%) had at least 1 AE that led to early discontinuation of the patient from the study; the majority of these were considered probably related to study medication.

No clinically significant changes in any of the other safety measures occurred during the study. The most commonly used concomitant medications were the anti-inflammatory dexamethasone (n = 55, 80.9%), the antiemetic metoclopramide (n = 36, 52.9%), and the diuretic furosemide (n = 25, 36.8%).

## Discussion

Chronic cancer pain is a highly prevalent condition. Although opioid analgesics are known to be effective for chronic moderate-to-severe pain in the short-term, data on their long-term use is more limited. Understanding the effects of long-term exposure to opioids has become particularly important in recent years as the life expectancy of cancer patients increases owing to improved oncological treatments.

CR opioid formulations are advocated for the management of chronic cancer pain because they can provide more consistent, around-the-clock pain relief with decreased dosing frequency (once- or twice-daily). OROS^® ^hydromorphone may be particularly well suited to the long-term management of cancer pain because it provides consistent plasma concentrations, sustained analgesia, and convenient once-daily dosing. In this open-label, single treatment extension study, OROS^® ^hydromorphone was administered to patients with moderate-to-severe chronic cancer pain who had successfully completed a previous short-term equivalence study and whose pain was controlled with a stable dose of medication. Prior opioid therapy in the previous equivalence study (OROS^® ^hydromorphone versus CR morphine) did not affect clinical outcomes such as efficacy or safety in this study.

The results demonstrate that pain control achieved with OROS^® ^hydromorphone is maintained for up to 1 year with repeated once-daily dosing in patients with chronic cancer pain. This was shown using a variety of measures, including the BPI items pain at its worst, pain at its least, pain on average, current pain, and pain relief, and patient and investigator global evaluations of overall treatment effectiveness. Throughout the study, pain scores were maintained at mild to moderate levels. These results support short-term studies of OROS^® ^hydromorphone for chronic cancer pain [20-22,34]. In addition, the degree to which patients' cancer pain interfered with their general activity, mood, ability to walk, work, relationships, sleep, and enjoyment of life was also maintained, suggesting that there was no worsening of QoL during the year. This has been demonstrated before; previous studies have shown a positive impact on QoL with CR opioid formulations [11,48]. There were no clear differences in the results when comparing patients who had received OROS^® ^hydromorphone or CR morphine in the previous equivalence study. Although most efficacy measures were maintained at similar levels throughout the study, for most measures, the mean scores were slightly worsened at end point compared with baseline. However, owing to the progressive nature of the disease, some deterioration is to be expected.

The most commonly reported AEs, with an incidence of at least 10%, were nausea, constipation, vomiting, anaemia, peripheral oedema, dyspnoea, asthenia, disease progression, somnolence, and urinary tract infection. These are events typically seen with the use of strong opioids or in a chronic cancer pain population. Slightly more AEs overall and AEs considered related to study treatment were reported in patients who had previously received OROS^® ^hydromorphone compared with CR morphine in the equivalence study; however, the clinical significance of this difference is questionable because of the degree and nature of AEs expected in an advanced cancer population. In addition, the majority of AEs were mild or moderate in severity. 19 patients died either during or after the study. There were slightly more deaths in patients treated with OROS^® ^hydromorphone compared with morphine in the previous equivalence study; however, all deaths were considered unrelated or unlikely to be related to study treatment. The occurrence of these deaths was not unexpected given the severity of patients' conditions and the progressive nature of the disease. Of the 32 patients who reported other SAEs, the majority of the events were considered unrelated or unlikely to be related to study treatment. 8 patients had SAEs that were considered to have a possible, probable, or definite relationship to study treatment. 9 patients reported AEs that led to early discontinuation from the study, and most of these AEs were considered probably related to study treatment. In conclusion, OROS^® ^hydromorphone was found to be safe and reasonably well tolerated in this extension study.

There are a number of limitations of this study, which may affect the interpretation of the results. This was an open-label, uncontrolled study, so the results cannot be directly compared to either no therapy or other opioid therapies. A large number of patients (58/68; 85.3%) did not complete the study. However, this is not unexpected given the severity and progressive nature of the disease; in fact, a large number of patients did not complete the study owing to death (n = 15) and progression of disease (n = 14). Dropouts due to lack of efficacy were uncommon (n = 8), but were to be expected given the progression of cancer. A further limitation was that no formal statistical analyses were done on the data. This was an open-label, single treatment arm trial mainly assessing the safety of long-term usage and secondary maintenance of efficacy; therefore, all analyses were done descriptively. End point was calculated using the LOCF method, a method that involves extrapolating the last observed measurement for the patient. This method was necessary because the study involved multiple visits and a critically ill patient population, and therefore a high number of dropouts was expected.

In spite of these limitations, this study has provided useful insights into the effectiveness of the long-term use of OROS^® ^hydromorphone for relief of cancer pain, which may be applicable to clinical practice. It also suggests that conversion from previous opioid therapy to OROS^® ^hydromorphone is feasible without loss of pain control. The effective morphine to hydromorphone conversion ratio varies from 4:1 to 8:1 in different publications [9,33,49-53]. A 5:1 morphine equivalents to hydromorphone conversion ratio is most often cited in the literature [21,22,34,43-46] and was found to be clinically useful in this study. The study population represents a rather small selected subgroup of patients, i.e. with advanced cancer and moderate-to-severe chronic cancer pain; nevertheless, the analgesia provided by OROS^® ^hydromorphone is maintained to some extent for up to a year, and the study will be useful in the development of other long-term studies of OROS^® ^hydromorphone.

## Conclusion

The results of this open-label, single treatment, extension study shows that long-term treatment with OROS^® ^hydromorphone is beneficial in the management of persistent, moderate-to-severe pain in patients with cancer.

## Competing interests

ALZA Corporation, that manufactures the drug, funded the current study; Johnson and Johnson Pharmaceuticals also contributed some funds for the editing of the current manuscript. MH was a paid advisor for Janssen-Cilag.

## Authors' contributions

MH and AT were investigators in this study and were involved in revising this manuscript for important intellectual content. JT contributed to the analysis of the study and reviewed the manuscript.

## Pre-publication history

The pre-publication history for this paper can be accessed here:


